# Intergenerational Associations of Chronic Disease and Polycystic Ovary Syndrome

**DOI:** 10.1371/journal.pone.0025947

**Published:** 2011-10-04

**Authors:** Michael J. Davies, Jennifer L. Marino, Kristyn J. Willson, Wendy A. March, Vivienne M. Moore

**Affiliations:** 1 Discipline of Obstetrics and Gynaecology, Robinson Institute, The University of Adelaide, Adelaide, Australia; 2 Discipline of Public Health, The University of Adelaide, Adelaide, Australia; State Key Laboratory of Reproductive Biology, Institute of Zoology, Chinese Academy of Sciences, China

## Abstract

**Background:**

Polycystic ovary syndrome (PCOS) is a common female endocrine disorder of heterogeneous clinical presentation, high disease burden, and unknown aetiology. The disease and associated conditions cluster in families, suggesting that PCOS may be the reproductive consequence of underlying chronic disease susceptibility.

**Objective:**

To determine whether parents of young women with PCOS were more likely to have a history of diabetes or cardiovascular disease in later adult life.

**Design, Setting and Participants:**

Structured interviews with 715 members of a cohort constructed by tracing female infants born at a single general hospital in Adelaide between 1973 and 1975. Participants were asked whether they had a pre-existing medical diagnosis of PCOS, and whether each parent had ever had high blood pressure, high cholesterol, diabetes, stroke, or heart disease. Maternal high blood pressure during pregnancy was taken from the medical record of the pregnancy with the study participant.

**Results and Conclusions:**

Mothers of women with PCOS were more likely than mothers of other women to have any cardiovascular disease (RR 1.78, 95% CI 1.29, 2.47), and nearly twice as likely to have high blood pressure (RR 1.95, 95% CI 1.38, 2.76). Fathers of women with PCOS were more than twice as likely to have heart disease (RR 2.36, 95% CI 1.44, 3.88) and over four times as likely to have had a stroke (RR 4.37, 95% CI 1.97, 9.70). Occurrence of cardiovascular disease in both mother and father are associated with the risk of PCOS in daughters. Further detailed study is required to elucidate the precise pathways that may be causally related to the observations.

## Introduction

Polycystic ovary syndrome (PCOS) is one of the most common endocrine disorders in women, affecting between 8 and 18% of the female population, depending on the diagnostic criteria used [1]. It has a heterogeneous clinical presentation including menstrual dysfunction, high androgen levels and evidence of multiple cysts within the ovary on ultrasound. The burden of disease is high, with complications including anovulatory infertility, endometrial cancer, hyperlipidemia, obesity and non-insulin dependent diabetes mellitus (NIDDM) [2]. Gestational diabetes appears to be common independent of prepregnancy weight and pregnancy weight gain [3], [4], [5]. The annual cost of evaluating and treating the disorder in the United States has been estimated at $4.36B (2005 USD) [6].

The aetiology of PCOS is unknown, although both genetic and intra-uterine factors, such as androgen exposure during fetal development, have been proposed [7]. There is evidence of clustering of the condition within families [8], although examination of data from twins does not support a simple pattern of inheritance [9]. There is also evidence of clustering for related chronic disease entities, including diabetes mellitus [10], [11] and cardiovascular disease risk [2], [12], [13], [14], within families in which PCOS is represented. This raises the question as to whether PCOS may be re-conceptualised as the reproductive consequences of underlying chronic disease susceptibility that is clustered within families. There is limited data on this matter, as there are few long-term follow-up studies of women with PCOS to examine disease progression, and none that have sought to compile detailed family histories within a population based sample.

The aim of the present study was to determine whether parents of women with an existing diagnosis of PCOS were more likely than other parents to have a history of diabetes or cardiovascular disease in later adult life.

## Methods

### Participants

As described elsewhere [1], the present cohort was built by tracing women born at the Queen Elizabeth Hospital (QEH) between 1973 and 1975. Daughters were initially traced after locating and contacting their mothers. If unsuccessful, attempts were then made to contact the daughter by other means, such as using electoral roll information. From 2199 birth records, 2046 (93.0%) daughters were traced, of whom 62 were deceased or disabled (3.0%), leaving 1984 (90.2%) women invited to participate in the study. Of these, 945 (47.6%) agreed to participate; of these, 715 were resident in Adelaide and thus eligible for the current analysis. Of those, twenty-two were estranged from their fathers and four from their mothers, and had no medical information about that parent. Estranged parents were excluded from analyses. There were no differences in birth outcomes between participants and non-participants. With regard to parents, and their daughters, the only evidence of difference between participants and non-participants was the slight over-representation of residents from the least socio-economically deprived suburbs [1].

### Study Protocol

Interviews were conducted by trained research nurses, usually in the home of the participant. A medical history was obtained, focusing on gynaecological history. The study was approved by the Central Northern Adelaide Health Service Ethics of Human Research Committee and The University of Adelaide Human Research Ethics Committee, and all participants gave written informed consent.

PCOS status was ascertained by participants' report of a pre-existing medical diagnosis. As women who had remained in Adelaide had greater access to specialists for diagnosis of PCOS than those who had moved elsewhere in the state, analyses presented in the current paper were limited to Adelaide residents. Analyses performed with all women were consistent with those limited to Adelaide residents. Parental illness was determined by asking participants whether each parent had ever had a medical history of high blood pressure, high cholesterol, diabetes, stroke, or heart disease. If a parent had ever had high blood pressure, stroke, or heart disease, he or she was classed as having had “any cardiovascular disease”. The date for the diagnosis of the conditions was not recorded. Maternal high blood pressure during pregnancy was taken from the medical record of the pregnancy with the study participant, and was defined as ever having had a visit with a recorded diastolic blood pressure in excess of 89 mmHg. Oral glucose tolerance testing in pregnancy was not performed routinely in the 1970s and was therefore unavailable for the present analysis to assess gestational diabetes.

### Statistical analysis

We used multivariate log-binomial regression to generate relative risks with daughter PCOS as the independent variable. Each parental condition became a dependent variable in separate regression models. Socioeconomic status (SES) was included in all models as potential confounders. The SES measure used was the Australian Bureau of Statistics Socioeconomic Indexes for Areas (SEIFA) quartile of disadvantage for the postal code of the family's residence at the time of the daughter's birth [15]. Parental age was not considered as a potential confounder. While associated with the outcomes of interest, we do not known of an association of parental age at delivery and risk of PCOS in daughters. Moreover, inclusion of parent age as a confounder in the analysis would not be appropriate if parental age was in the causal pathway. This would occur if there is an association between risk of chronic disease and age at delivery, which is plausible in the case of maternal history of diabetes.

Models used log-binomial rather than logistic regression because where outcomes are common in a study population (>5%), as with some parental conditions considered, odds ratios may underestimate relative risk (for a discussion, see [16]). Missing values for maternal smoking during pregnancy (n = 41), maternal high blood pressure during pregnancy (n = 283), maternal disease status (n = 24) and paternal disease status (n = 100) were multiply imputed using regression models, with 100 imputations. Sensitivity analyses were performed by varying the multiply imputed models. The results from the sensitivity analyses were similar to those presented in this paper.

A *P* value of <0.05 for the Wald test on the adjusted estimate of interest was considered statistically significant. All statistical analyses were carried out using SAS® software, Version 9.2 of the SAS System for Windows. (2009 SAS Institute Inc., Cary, NC, USA.)

## Results

Among women residing in Adelaide, 41 (5.7%) reported an existing diagnosis of PCOS (Table 1). Nearly all mothers survived to the time of interview, as did most fathers.

**Table 1:** Demographic characteristics of parents of young women.

	Mother of PCOS daughter (n = 41) Number (%)	Mother of unaffected daughter (n = 674) Number (%)	Father of PCOS daughter (n = 41) Number (%)	Father of unaffected daughter (n = 674) Number (%)
**Parents living at time of interview n (%)**	40 (97.6)	648 (96.1)	38 (92.7)	597 (90.7)
**Mother's age at time of interview, median, midquartile range (years)**	52, 49–56	54, 51–58	–	
**Parents known to be estranged n (%)**	0 (0.0)	4 (0.6)	0 (0.0)	22 (3.4)
**Household SEIFA at birth (median, midquartile range)**	975.8 873.9–1033.7	947.0 893.3–1008.5	975.8 873.9–1033.7	947.0 893.3–1008.5
**Mother's country of birth n (%)**				
Australia	28 (68.3)	405 (60.1)	–	–
United Kingdom	4 (9.8)	69 (10.2)	–	–
Other Northwest Europe	1 (2.4)	28 (4.2)	–	–
Southeast Europe	8 (19.5)	158 (23.4)	–	–
Other	0	14 (2.1)	–	–
**Education n (%)**				
Some primary school	3 (7.3)	49 (7.3)	4 (9.8)	67 (10.0)
Completed primary school	1 (2.4)	42 (6.2)	0 (0.0)	41 (6.1)
Some high school	24 (58.5)	276 (41.0)	14 (34.1)	169 (25.1)
Completed high school	3 (7.3)	75 (11.1)	2 (4.9)	43 (6.4)
Trade school/non-university	7 (17.1)	150 (22.2)	13 (31.7)	213 (31.7)
Any university	2 (4.9)	40 (5.9)	4 (9.8)	42 (6.3)
Don't know	1 (2.4)	41 (6.1)	4 (9.8)	97 (14.4)
**Smoked while pregnant n (%)**	7 (18.9)	156 (24.5)		
**Parity n (%)**				
0	20 (48.8)	285 (42.3)		
1	12 (29.3)	220 (32.6)		
2	5 (12.2)	112 (16.6)		
3+	4 (9.8)	57 (8.4)		
**Mother's age at delivery median, midquartile range (years)**	22, 19–26	24, 21–28		

PCOS: Polycystic ovary syndrome; SEIFA: Socioeconomic Indexes for Areas.

Table 2 shows relationships between parental chronic disease status and daughter's PCOS. Mothers of women with PCOS were more likely than other mothers to have a daughter report of any cardiovascular disease (adjusted relative risk (RR) 1.78 (95% confidence interval (CI) 1.29, 2.47), and were nearly twice as likely to have high blood pressure (RR 1.95, 95% CI 1.38, 2.76). Mothers of women with PCOS were also more likely to have had high blood pressure during pregnancy (RR 1.49, 95% CI 0.97, 2.30), although this relationship did not reach statistical significance. There was no statistically significant relationship between daughter's PCOS and reported maternal hypercholesterolemia, diabetes, heart disease or stroke.

**Table 2:** Relationships between parental health condition and PCOS in young women.

Health Condition	PCOS cases N (%)	Unaffected N (%)	Unadjusted RR^1^ (95% CI)	Adjusted RR^2^ (95% CI)
**Mother**	41 (100.0)	674 (100.0)	–	–
High blood pressure	20 (48.8)	172 (25.5)	**1.91 (1.36, 2.69)**	**1.95 (1.38, 2.76)**
High cholesterol	8 (19.5)	139 (20.6)	0.95 (0.50, 1.80)	0.92 (0.48, 1.76)
Diabetes	5 (12.2)	64 (9.6)	1.28 (0.54, 3.00)	1.37 (0.58, 3.22)
Stroke	2 (4.9)	25 (3.7)	1.31 (0.32, 5.36)	1.27 (0.30, 5.31)
Heart disease	4 (9.8)	31 (4.6)	2.13 (0.79, 5.78)	2.07 (0.76, 5.60)
High blood pressure during pregnancy	18 (44.9)	204 (30.3)	1.47 (0.96, 2.27)	1.49 (0.97, 2.30)
Any cardiovascular disease^3^	21 (51.2)	196 (29.1)	**1.76 (1.28, 2.43)**	**1.78 (1.29, 2.47)**
**Father**	41 (100.0)	674 (100.0)		
High blood pressure	11 (26.5)	200 (29.7)	0.89 (0.51, 1.54)	0.90 (0.52, 1.55)
High cholesterol	15 (36.1)	219 (39.2)	1.11 (0.71, 1.74)	1.10 (0.70, 1.72)
Diabetes	8 (19.5)	106 (15.7)	1.26 (0.64, 2.51)	1.28 (0.65, 2.53)
Stroke	8 (19.5)	28 (4.2)	**4.45 (1.97, 10.01)**	**4.37 (1.97, 9.70)**
Heart disease	14 (13.2)	101 (15.0)	**2.27 (1.38, 3.74)**	**2.36 (1.44, 3.88)**
Any cardiovascular disease^3^	20 (48.8)	264 (39.2)	1.24 (0.88, 1.76)	1.29 (0.88, 1.96)

PCOS: polycystic ovary syndrome; RR: relative risk; CI: confidence interval. Sig. associations in bold.

1 Combined results of multiply imputed models.

2 Adjusted for SEIFA at birth. Combined results of 11 multiply imputed models.

3 Any cardiovascular disease: high blood pressure, stroke or heart disease.

Fathers of women with PCOS were more than twice as likely to have a report of heart disease (RR 2.36, 95% CI 1.44, 3.88) and over four times as likely to have had a stroke (RR 4.37, 95% CI 1.97, 9.70. Among fathers, there was no significant relationship between reported hypercholesterolemia, diabetes or high blood pressure and daughter's PCOS.

Findings were unchanged by adjustment for socioeconomic status. Secondary analyses with all participants were consistent with the present findings, as were sensitivity analyses.

## Discussion

In this family history study, we confirmed previous associations indicating that the parents of women who had been diagnosed with PCOS were more subject to chronic diseases than parents of women who had never been diagnosed with PCOS [17]. Specifically, high blood pressure in mothers was nearly twice as frequent if a daughter had PCOS. This finding was consistent with, and more robust than, the association between mother's elevated blood pressure during pregnancy as obtained from the maternity record and daughter's PCOS. In addition, the relative risks for heart disease, diabetes and stroke were also elevated but not significant.

Interestingly, the pattern of maternal disease was replicated, in part, in the father's history, with particularly robust associations for stroke and heart disease. Again, the associations with parental history of diabetes were elevated but the wide confidence intervals precluded directly replicating the findings reported previously [11].

This study varied from those reported previously as it is a community based cohort using a defined sampling frame, rather than a convenience sample from clinics. Most other studies used infertility or gynaecology clinic samples as the source of both PCOS and comparison populations, which is problematic as the population of probands with infertility problems might reasonably be expected to have higher proportions of both sex hormone and metabolic problems than would a general population of women, producing a comparison family population more similar to the PCOS families than a general population of families. This results in both underestimation of associations and loss of generalizability. The attenuation of measures of association may account for some of the inconsistent findings in previous family studies.

Our participation rate was high, given the 30+ years elapsed before tracing, and showed little evidence of participation bias [1]; the study also used trained nurse-interviewers. However, it did not include direct assessment of disease status in either daughter or parents, except for the use of hospital records to ascertain maternal high blood pressure in pregnancy.

Nevertheless, there is merit in these proxy reports on disease experience as it has been demonstrated previously [18] that CHD family history can be captured effectively with accuracy over 80%. The additional imprecision from recall and reporting, and limited sample size within the outcome groups is likely to have increased the confidence intervals observed here, which may explain why a number of the associations did not achieve statistical significance. We therefore do not preclude other potential associations, including the observed family history of diabetes in both mother and father. Further, while the study relied upon daughter's reports of parental disease history, the consistency of effect for maternal high blood pressure from daughter reports and independent maternity records suggests that the reports are not spurious. Similarly, if reporting bias were the major contributor to the findings, then one would expect strong and specific associations for a family history of diabetes, which has a well-established association with PCOS.

Replication of these observations in other cohorts is warranted, as is detailed clinical examination of the surviving parents, which was beyond the scope of the current project that focussed primarily on reproductive experiences of young women.

Parent-of-origin effects have been noted previously for CVD risk in fathers, including incident stroke, and metabolic perturbations in offspring [12], [19]. For example, in a case-control study of PCOS Moini observed that a maternal family history of diabetes was not associated with PCOS, in contrast to a paternal family history of heart attack and thrombosis. [19].

The association between paternal stroke and PCOS may be associated with some direct mechanism, or mediated through a factor(s) associated both with stroke and PCOS risk. Obesity is one such factor, as paternal obesity has been associated with PCOS [20]. This is of potential interest as recent experimental observations in a rodent model reveal that paternal high fat diet and increased body weight prior to conception induced epigenetic changes in female offspring with features of impaired insulin secretion and glucose tolerance that worsened with time [21]. Further, the demonstration that paternal high fat diet altered the expression of pancreatic islet genes in adult female offspring provides evidence of biological plausibility for non-genetic, intergenerational transmission of metabolic features from father to daughter.

In conclusion, these findings highlight the presence of robust associations at an observational level that invoke a range of biologically plausible mechanisms and indicate a need for further detailed clinical investigation in both parents and child.
